# Sympathy for the Erring

**Published:** 1875-11

**Authors:** 


### SYMPATHY FOR THE ERRING.

Of how much of our indignation against even a deliberate wrong would we be disarmed, if we could but know for ourselves a tithe of all the sorrow, and trouble, and disappointment, the poor erring heart has passed through! What efforts were made in youth to stand up against the pressure of the world, and how, when fallen, from miscalculation, or an over-confiding nature or want of tact, it bravely rose up and tried again; and when hard necessity came and drove it to the wall, how it looked around for help, and waited, still striving to stand upright, and fell while striving; and even when fallen, how it yearned for one more chance to rise and be a man, how loth at last, to give up all for lost!—could we but see a thousandth part of these struggles, as they rend our brother's bosom and almost break his heart, how should it disarm us of our vindictiveness, and incline us even to run to him, and raise him up, and stand by him, and, with godlike forgiveness, bid him, in tones of encouragement, "Try, try again!"

---

Wasted Time.—Lost wealth may be restored by industry; the wreck of health regained by temperance; forgotten knowledge restored by study; alienated friendship smoothed into forgetfulness; even forfeited reputation recovered by penitence; but who ever again looked upon his vanished hours?—recalled his slighted years?—stamped them with wisdom?—or effaced from heaven's record the fearful blot of wasted time?

---

Faith in our own ability is half of every battle.

